# Insights into the Gut Microbial Diversity of Wild Siberian Musk Deer (*Moschus moschiferus*) in Republic of Korea

**DOI:** 10.3390/ani14203000

**Published:** 2024-10-17

**Authors:** Nari Kim, Kyung-Hyo Do, Chea-Un Cho, Kwang-Won Seo, Dong-Hyuk Jeong

**Affiliations:** 1 College of Veterinary Medicine, Chungbuk National University, Cheongju 28644, Republic of Korea; queng91@gmail.com (N.K.); pollic@chungbuk.ac.kr (K.-H.D.); vetskw16@cbnu.ac.kr (K.-W.S.); 2 Yanggu Goral/Musk Deer Conservation Center, Yanggu 24506, Republic of Korea; goralmusk71@korea.kr; 3 Wildlife Center of Chungbuk, Cheongju 28116, Republic of Korea

**Keywords:** fecal microbiome, *Moschus moschiferus*, Siberian musk deer, non-invasive, 16S rRNA, wildlife conservation

## Abstract

**Simple Summary:**

The Siberian musk deer is a vulnerable species, and understanding its health is crucial for conservation. This study investigated the gut microbiome of wild Siberian musk deer in three different locations in Republic of Korea. By analyzing fecal samples through 16S rRNA sequencing, we observed significant differences in the alpha diversity among Siberian musk deer from various habitats, while the beta diversity showed no significant differences. Despite the lack of variation in the beta diversity, we identified unique bacterial compositions specific to both the species and the different locations. These insights are valuable because the gut microbiome plays a vital role in animal health, and the differences in microbial communities can be influenced by factors such as diet and habitat. This study is the first to provide important information about the gut microbiota of wild Siberian musk deer, which could aid in developing future conservation strategies.

**Abstract:**

The gut microbiota plays a crucial role in the health and well-being of wildlife. However, its composition and diversity remain unexplored, particularly in threatened species such as the Siberian musk deer (SMD). This study aimed to elucidate the gut microbiota composition within different wild SMD communities for assessing their health status. We conducted the first comprehensive fecal microbiome analysis of wild SMD inhabiting three distinct locations in Gangwon Province, Republic of Korea (Korea). Fecal samples were collected non-invasively and 16S rRNA gene sequencing was performed for gut microbiota characterization. Consistent with previous research, Firmicutes and Bacteroidetes were the dominant phyla in the gut microbiota of wild SMD. Planctomycetota was a prevalent phylum in wild SMD gut microbiota, warranting further investigation of its ecological significance. While significant differences were observed in the gut microbiota richness among the three groups, no significant disparities were detected in the beta diversity. Additionally, certain genera exhibited distinct relative abundances among the groups, suggesting potential associations with geographic factors, gut disorders, and dietary habits. Our findings provide valuable insights into the gut microbiome of wild SMD and offer a foundation for future microbiome-based conservation efforts for this vulnerable species.

## 1. Introduction

Siberian musk deer (*Moschus moschiferus*; SMD) belong to the ungulate family Moschidae and are distributed across Korea, China, Russia, Mongolia, Kazakhstan, Kyrgyzstan, Nepal, and Vietnam [1]. Musk produced by the male musk deer to attract females has been extensively used in Chinese medicine and perfume manufacturing for more than 5000 years [2]. Despite hunting prohibitions, wild *Moschus* populations have declined since the twentieth century due to illegal poaching, habitat fragmentation, and other human-induced activities [3], leading to their vulnerable status on the IUCN Red List.

In Republic of Korea (Korea), the number and distribution area of SMD have sharply decreased from the 1950s to 1999 [4], with less than 40 animals being reported [5]. To conserve the species, it is classified as a Class I Endangered species by the Korean Ministry of Environment and designated as Natural Monument No. 216 by the Cultural Heritage Administration of Korea. Although recent camera trapping efforts have successfully captured some SMD, the precise number and habitat locations within the country are unknown. Despite the significance of species conservation, information acquisition is severely constrained by the small population size and cautious behavior of SMD in the wild.

Fecal microbiome analysis, a non-invasive technique, is widely used in wildlife conservation because of its efficient and cost-effective management while addressing public expectations for animal welfare [6]. Microbiomes reflect and influence the interactions between the host organism and its environment, thereby affecting host health [7]. The gut microbiota is shaped by various factors such as the habitat environment [8], diet [9], age [10], seasonal change [11], disease [12], and reproduction [13]. These factors allow for the direct or indirect assessment of the host through fecal microbiome analysis. Although fecal samples may not capture the complete dynamics of bacterial populations across the entire gut, they reflect the composition of the overall intestinal microbiome [14]. Therefore, understanding the composition and distribution of individual microbiota can aid in appropriate individual management and wildlife conservation policies [15].

Numerous studies have explored the composition and diversity of the gut microbiota in various musk deer species. Bacterial diversity varies between the forest musk deer (*Moschus berezovskii*; FMD) and SMD. Su et al. [2] identified significant taxonomic differences, noting a higher relative abundance of Firmicutes in SMD than in FMD. Additionally, Alpine musk deer (*Moschus chrysogaster*; AMD) and FMD exhibit distinct microbial compositions, with a higher diversity observed in FMD than that in AMD, along with differences in dominant species [16,17]. The fecal microbiome has also been compared between captive and wild FMD [18] and AMD [19,20,21]. Li et al. [18] has revealed significant differences in the relative abundances of Firmicutes and Bacteroidetes between captive and wild FMD, with a higher Firmicutes-to-Bacteroidetes ratio (F/B ratio) observed in wild FMD than in their captive counterparts. Sun et al. [19] has reported similar findings in gut microbiota comparisons between captive and wild AMD and observed a higher F/B ratio in wild AMD compared to the captive species. Moreover, wild AMD may be less susceptible to intestinal diseases and maintain a more stable intestinal structure than the captive populations [20], suggesting that distinct environmental conditions influence the deterministic assembly of microbial communities [21]. Studies have also been conducted on the factors influencing the gut microbiota within captive populations of the same species of musk deer. The social behavior in captive SMD can influence the diversity of the gut microbiome [22]. Differences in location and the migration of captive AMD significantly affect the relative abundance of intestinal microbial species [23]. In captive FMD, the gut microbiome structure is affected by various factors such as location during ex situ conservation [24], sex, age [25], and season [26], as well as diseases such as pneumonia and abscesses [27,28], probiotic supplementation [29,30], and the chemical components of musk [31]. However, most studies on musk deer gut microbiota have focused on captive populations in breeding centers, with limited research conducted on wild musk deer focusing primarily on FMD and AMD [18,19,20,21].

Although prior studies have provided crucial insights into the gut microbiome status and its relationship to different factors in musk deer, such an analysis has not yet been conducted in wild SMD. This is essential since the microbial composition between wild and captive SMD may differ owing to various factors, such as diet and habitat. Considering the limited number and careful nature of wild SMD, the fecal microbiome analysis can serve as an efficient non-invasive method to protect the species from harm while providing valuable and informative data.

Furthermore, biogeography significantly influences the gut microbiome composition in various animals, including Rocky Mountain elk, house mice, red squirrels, and North American moose [8,32,33,34]. These results underscore the importance of analyzing microbiome differences in hosts across spatial distances. Consequently, microbiome analyses that consider geographic factors within SMD species are warranted.

Hence, the aim of this study was to enhance the understanding of the gut microbiome in different wild SMD communities and to offer a scientific benchmark for assessing their health status. To achieve this, we collected wild SMD fecal samples from three different sites in Gangwon Province, Korea during spring and performed a comparative analysis of the bacterial microbiome using 16S rRNA gene sequencing. These results will be useful for acquiring basic information on wild SMD and providing quantitative indices for long-term SMD conservation.

## 2. Materials and Methods

### 2.1. Sample Collection

**Figure 1:**
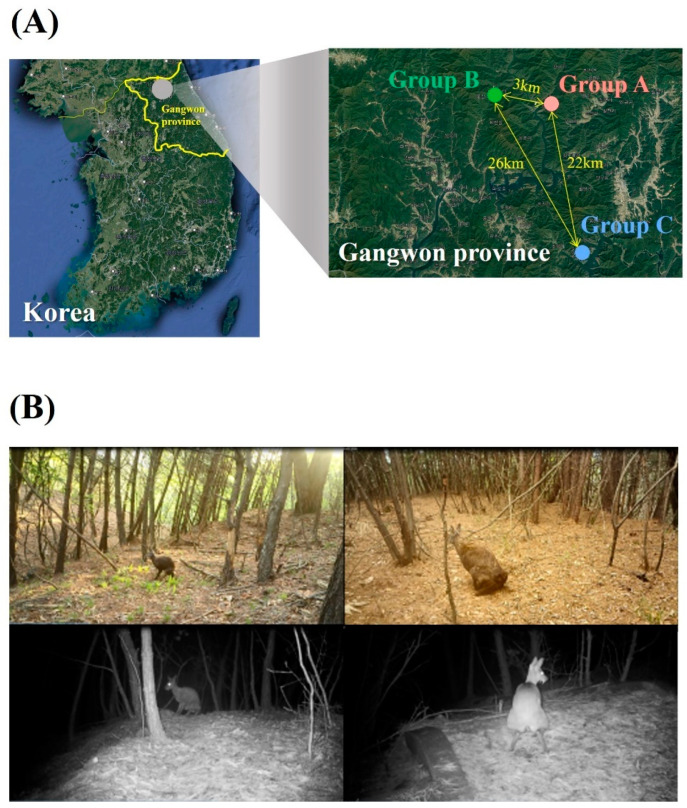
Sampling sites and captured images of wild Siberian musk deer (SMD). (**A**) The three distinct locations in Gangwon province for collecting wild SMD fecal samples. (**B**) Camera trapping confirmed the defecation of each individual wild SMD.

A total of 23 fresh fecal samples were collected in March and April 2023 from 16 different locations in Yanggu (Group A), 3 different locations in Hwacheon (Group B), and 4 different locations in Chuncheon (Group C), all located in the Gangwon province (Figure 1A). The maximum home range of SMD is known to be 300 ha (3 km^2^) [35], which is smaller than the linear distance between Groups A and B (3 km). Additionally, the presence of Peace Dam between Groups A and B, as well as the 38.9 km^2^ artificial Paroho lake separating Groups A, B, and C, further supports that the samples collected were from distinct musk deer populations. The locations of musk deer defecation were confirmed using unmanned sensor cameras, thereby confirming that all fecal samples belonged to wild SMD (Figure 1B). The number of collected fecal samples varied across the three groups, with Group A having significantly more defecation sites than Groups B and C. Collected fecal samples from the different locations within the same group may have been excreted by the same individual.

The altitudes of the sampling sites were approximately 685, 645, and 430 m in Group A, B, and C, respectively. The total average precipitation and mean temperature in Gangwon province during March to April 2023 were 6.6 mm and 9.7 °C, respectively, based on data from the nearest meteorological observation centers of the Korean Meteorological Administration. Fecal samples from wild SMD that appeared externally moist and sticky were preserved and collected.

Fresh feces were collected using sterile gloves and bags to prevent human contamination. All samples were stored in a refrigerator at −20 °C after labeling. Subsequently, the samples were transported to the laboratory and stored in deep freezer at −80 °C for one month until DNA extraction.

### 2.2. DNA Extraction and 16S rRNA Gene Sequencing

Fecal samples (200 mg) from musk deer were collected by slicing and using the inner portions under sterile conditions to minimize potential contamination. The samples were then transferred to 2 mL sterile centrifuge tubes. Bacterial DNA was extracted from each fecal sample using a Maxwell RSC Fecal Microbiome DNA Kit AS1700 (Promega, Madison, WI, USA), following the manufacturer’s instructions. DNA quality was assessed using a Qubit dsDNA HS assay kit (Life Technologies, Gent, Belgium). The DNA extracts were preserved at −80 °C until further processing.

The V3–V4 hypervariable region of the 16S rRNA gene was polymerase chain reaction (PCR)-amplified using the following primers, as recommended by Illumina’s protocol: forward 5′-TCGTCGGCAGCGTCAGATGTGTATAAGAGACAGCCTACGGGNGGCWGCAG-3′ and reverse 5′-GTCTCGTGGGCTCGGAGATGTGTATAAGAGACAGGACTACHVGGGTATCTAATCC-3′. Subsequently, a second PCR was conducted to attach barcodes using the Nextera XT Index Kit (Illumina, San Diego, CA, USA). Library quality was evaluated using a Qubit fluorometer (Thermo Fisher Scientific, Waltham, MA, USA). Finally, the high-throughput sequencing was performed on an Illumina MiSeq platform using the MiSeq Reagent Kit v3 (600 cycles) (Illumina).

### 2.3. Operational Taxonomic Unit and Taxonomy Assignments

The 16S rRNA gene sequences were obtained and processed using QIIME 2 2023.7 [36]. Initially, the sequences were demultiplexed and trimmed. Subsequently, forward and reverse reads were merged and matched to their respective samples based on their assigned barcodes. The joined sequences were subjected to quality filtering, and sequences that failed to meet specific criteria (sequence length <20 or >300 nucleotides) were discarded. The SILVA v138 99% full-length database was used to cluster the operational taxonomic units (OTUs) [37].

### 2.4. Bioinformatics and Statistical Analyses

The sequences were rarefied, before computing alpha and beta diversity statistics. Alpha diversity indices, including Abundance-based Coverage Estimator (ACE), Chao1, Shannon, and Simpson indices, were computed based on rarefied samples, to assess the richness and diversity within the bacterial community. Bray–Curtis distances were used to evaluate beta diversity, followed by principal coordinate analysis (PCoA), to visualize the differences in microbial diversity. A heatmap analysis was conducted to assess whether samples could be grouped based on habitats, using Bray–Curtis dissimilarity and average linkage hierarchical clustering. Linear discriminant analysis effect size (LEfSe) analysis was performed to assess the significance of differentially abundant features with *p* < 0.05, and a linear discriminant analysis (LDA) score > 2.0. All these tests were performed using MicrobiomeAnalyst (https://www.microbiomeanalyst.ca/, accessed on 28 November 2023) [38].

Considering the small sample size of the three groups (*n* in Group A = 13, *n* in B = 3, and *n* in C = 4), a non-parametric statistic, the Kruskal–Wallis H-test, was used to analyze the relative abundances of the five most abundant phyla and the nine most abundant genera among the groups. Statistical significance was set at *p* < 0.05. Analysis was performed using the SPSS software package version 28.0.0.0 (IBM Corp., New York, NY, USA). Dunn’s post hoc test was used to identify specific groups with significant differences. The relative abundance of the bacteria was visualized using R version 4.2.3 (https://www.r-project.org, accessed on 29 February 2024). Venn diagrams were used to visually identify shared OTUs among all three groups, employing Venny 2.1.0 (https://csbg.cnb.csic.es/BioinfoGP/venny.html, accessed on 18 February 2024). STAMP v 2.1.3 [39] was used, to visualize the differences in bacterial composition at the genus level among the groups, with *p* < 0.05.

## 3. Results

### 3.1. Validation of 16S rRNA Sequencing Results

**Figure 2:**
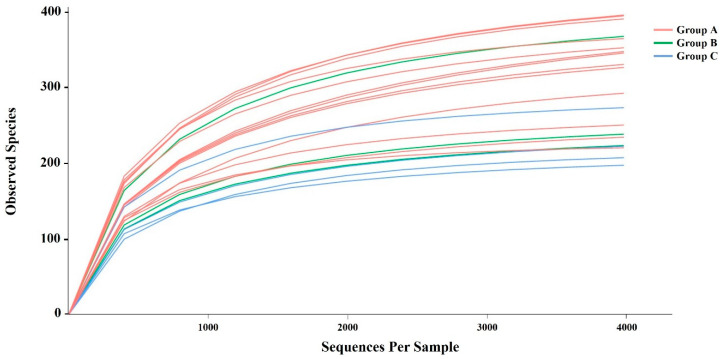
Rarefaction curves of observed species for the 20 SMD samples, with each curve color-coded according to the sampling locations. The X-axis represents the number of valid sequences per sample and the Y-axis denotes the observed species (operational taxonomic units, OTUs). As the sequencing depth increases, the observed species also increases and stabilizes with the expansion of extracted sequences, signifying an optimal point where the quantity of sequencing data is sufficient.

A total of 2,501,245 sequences (raw reads) were obtained from 23 SMD samples using Illumina MiSeq sequencing technology, resulting in 1,945,261 effective sequences (effective reads) after trimming using Qiime2. Among the 23 collected and sequenced samples, 3 exhibited notably low sequence counts (less than 100 sequences), suggesting possible degradation or contamination during collection. Consequently, these samples were excluded from the dataset before further analysis. The average number of effective sequences obtained per sample was 121,247, with counts ranging from 41,937 to 138,621. A total of 5279 OTUs were obtained after clustering the reads at 97% similarity. As the sequencing depth increased, the number of OTUs initially increased before plateauing at approximately 4000 reads (Figure 2), indicating adequate sequencing data for analysis. The OTUs were categorized into 9 phyla, 15 classes, 36 orders, 54 families, and 90 genera. Good’s coverage approached 99%, indicating that most bacterial features in our samples were identified.

**Figure 3:**
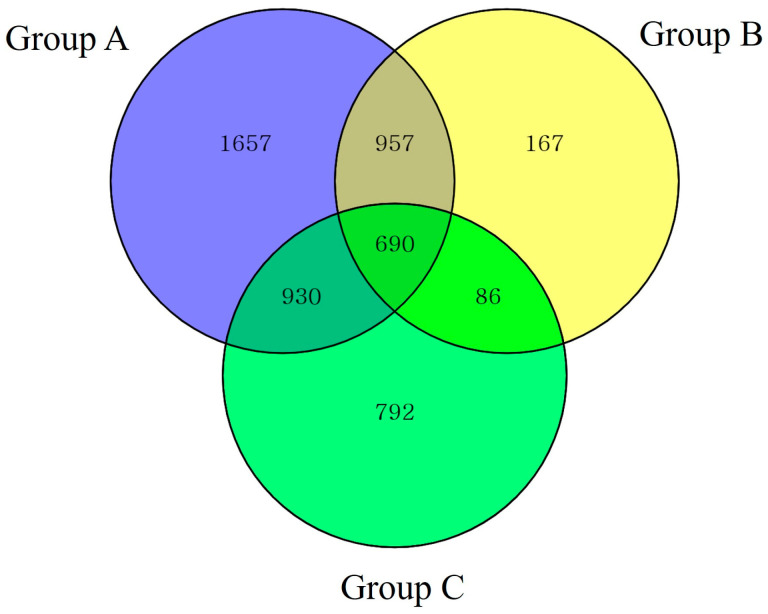
OTU Venn diagrams and bacterial taxa (phylum-level) pie charts.

Venn diagrams (Figure 3) illustrate the common and unique gut microbiota of SMD from three different locations (Groups A, B, and C) in Gangwon province. The bacterial populations shared among the SMD within each group were regarded as the core microbiota. A total of 690 OTUs were shared by all the groups: 957 between A and B, 930 between A and C, 86 between B and C, 1657 in A, 167 in B, and 792 in C.

**Figure 4:**
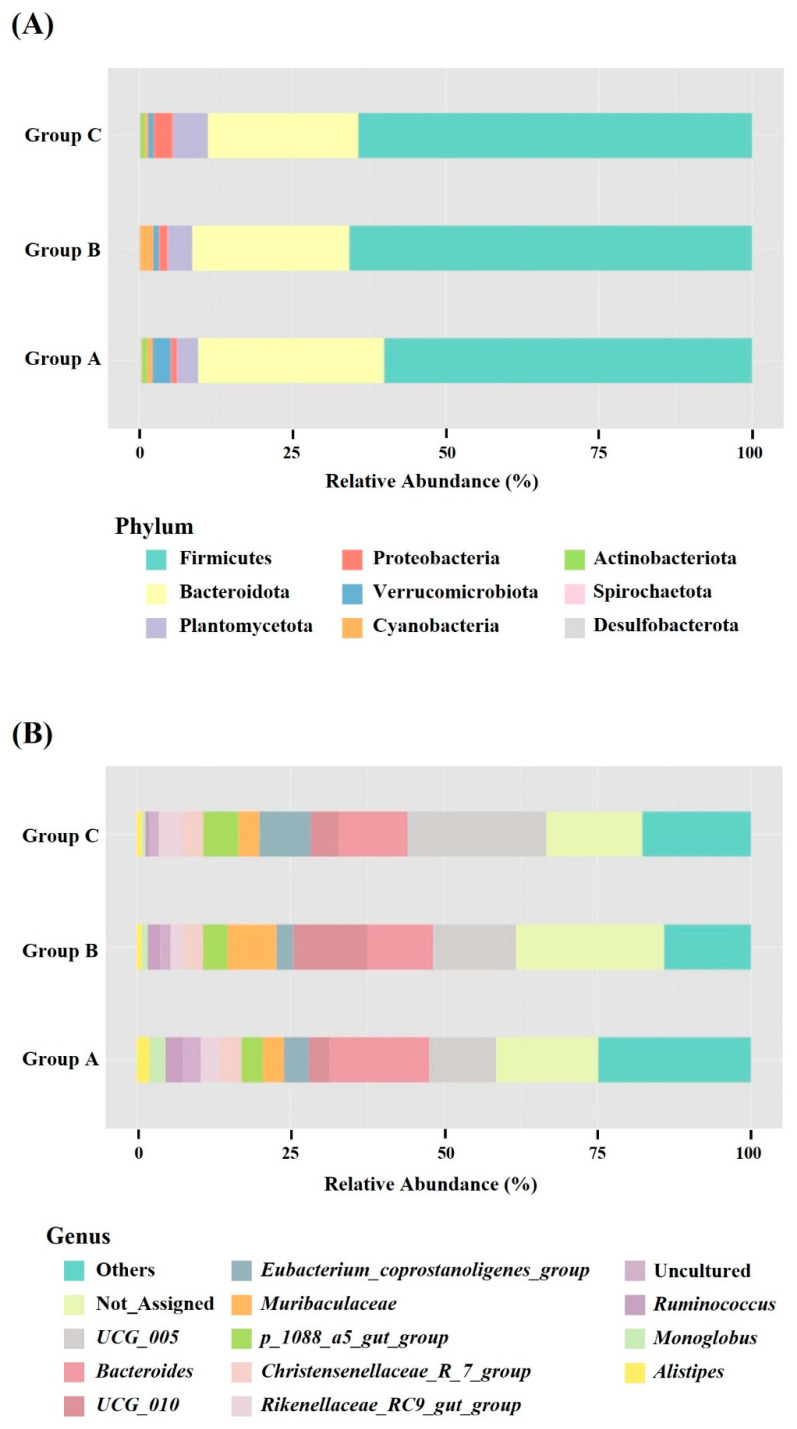
Bacterial compositions of SMD among three groups at the phylum (**A**) and genus (**B**) levels. The bar charts depict the average relative abundance of all phyla and the most prevalent genera identified in Groups A, B, and C.

Stacked bar charts were prepared at the phylum level, to illustrate the composition of the gut microbiota in wild SMD across different locations (Figure 4A) and genus levels (Figure 4B). The predominant phyla were Firmicutes (Group A, 59.34%; B, 65.82%; and C, 64.98%) and Bacteroidota (A, 30.78%; B, 25.72%; and C, 24.52%), followed by Planctomycetota (A, 3.58%; B, 4.11%; and C, 5.56%), Proteobacteria (A, 3.09%; B, 0.94%; and C, 0.98%), Verrucomicrobiota (A, 0.95%; B, 1.32%; and C, 2.82%), and Cyanobacteria (A, 0.81%; B, 1.80%; and C, 0.42%). The F/B ratios among the three groups (A, 2.30; B, 2.61; and C, 3.66) were not significantly altered, as determined by the Kruskal–Wallis H-tests (*p* = 0.323).

The most common genera in terms of relative abundance were *Bacteroides* (A, 16.37%; B, 10.15%; and C, 11.52%), *Oscillospiraceae*_*UCG_005* (A, 10.37%; B, 13.93%; and C, 22.00%), *Eubacterium_coprostanoligenes_group* (A, 3.98%; B, 2.84%; and C, 8.28%), *Oscillospiraceae*_*UCG_010* (A, 3.19%; B, 12.47%; and C, 4.52%), *Muribaculaceae* (A, 3.57%; B, 8.05%; and C, 3.35%), *p_1088_a5_gut_group* (A, 3.58%; B, 4.11%; and C, 5.56%), *Christensenellaceae_R_7_group* (A, 3.64%; B, 3.32%; and C, 3.54%), *Rikenellaceae_RC9_gut_group* (A, 3.38%; B, 2.42%; and C, 3.55%), *Ruminococcus* (A, 2.94%; B, 2.00%; and C, 1.89%), *Monoglobus* (A, 2.43%; B, 0.92%; and C, 0.60%), and *Alistipes* (A, 2.05%; B, 0.83%; and C, 0.86%). Not_Assigned represents sequences that could not be matched to any known taxonomic reference, comprising 16.96, 24.08, and 15.45% of Groups A, B, and C, respectively.

### 3.2. Analysis of Alpha and Beta Diversity

**Figure 5:**
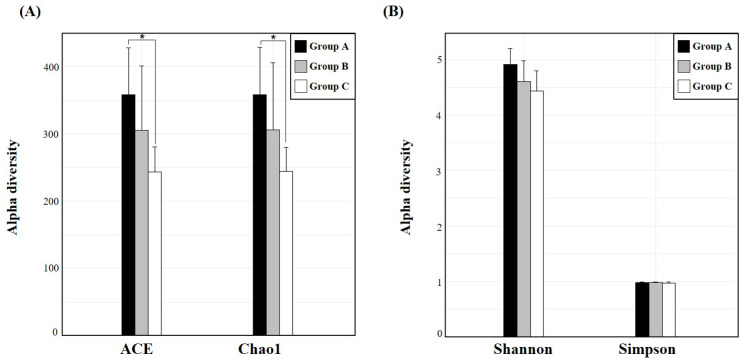
Bar diagrams depicting α-diversity indices of the gut microbiota among Groups A, B, and C. (**A**) The ACE and Chao1 indices were used to assess the number of OTUs within each community. (**B**) The Shannon and Simpson indices were used to estimate microbial diversity within each group. * represents *p* < 0.05.

We compared the α-diversity, which quantifies the richness and evenness within a single community, among fecal samples of SMD in three groups using different indicators. The ACE and Chao1 indices were employed to assess the total number of species, whereas the Shannon and Simpson indices were used to measure the species diversity. The ACE and Chao1 indices of the gut microbiome were significantly different among Groups A, B, and C (*p* < 0.05) (Figure 5A); however, no significant differences were observed in the Shannon and Simpson indices (*p* = 0.063 and 0.204, respectively) (Figure 5B). Supplementary Table S1 shows the detailed figures of α-diversity indices among different groups.

**Figure 6:**
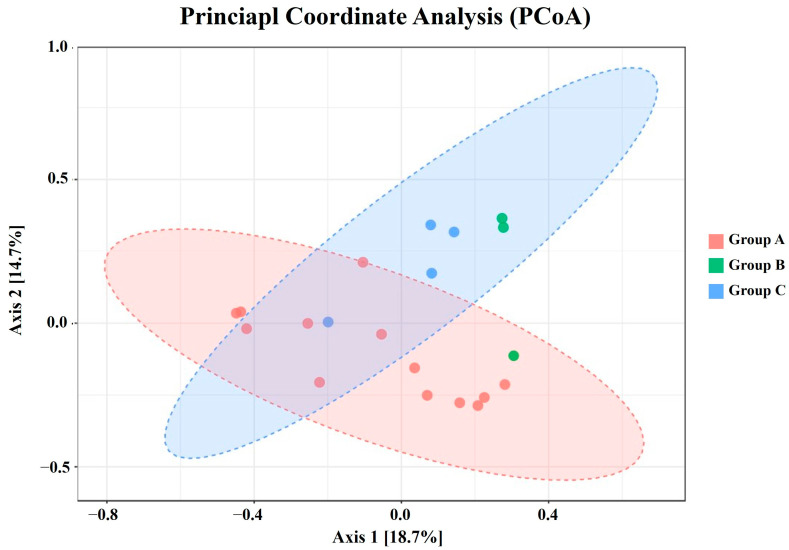
Principal coordinate analysis (PCoA) plot of β-diversity based on Bray–Curtis index. Statistical significance was determined using PERMANOVA. Samples from the same group are depicted in the same color, with the horizontal and vertical axes representing relative distances.

To assess the β-diversity, which reflects the differences in species diversity among groups, PCoA was visualized using the Bray–Curtis Index (Figure 6). The X and Y axes represent the selected dimensions and show the percentages of dissimilarities in the sample composition, with scales indicating relative distances. Although no significant differences were observed among the three groups due to the low coefficient of determination (*R*^2^ = 0.22; *F*-value: 0.22; and *p* < 0.05), the arrangement of Groups A and C displayed distinct clusters with slight overlaps.

### 3.3. Analysis of Differences in Gut Microbiota Composition

**Figure 7:**
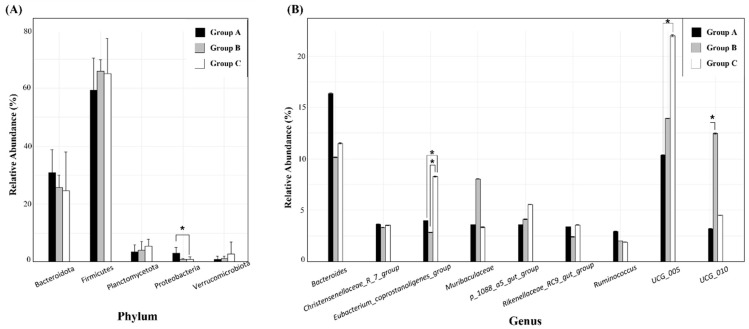
Bar diagrams displaying the relative abundances (mean % ± standard deviation) of (**A**) five major bacterial phyla and (**B**) nine major bacterial genera among Groups A, B, and C. * *p* < 0.05.

Kruskal–Wallis H-tests were used to analyze the microbial community composition among the three groups at the phylum and genus levels. At the phylum level, significant differences were observed only in the abundance of Proteobacteria, with Group A exhibiting a higher abundance than Groups B and C. At the genus level, the relative abundances of *Oscillospiraceae*_*UCG_005*, *Oscillospiraceae_UCG_010*, and *Eubacterium_coprostanoligenes_group* were significantly higher in Group C compared to Group A, in Group B compared to Group A, and in Group C compared to the other groups, respectively (Figure 7; Supplementary Table S2).

**Figure 8:**
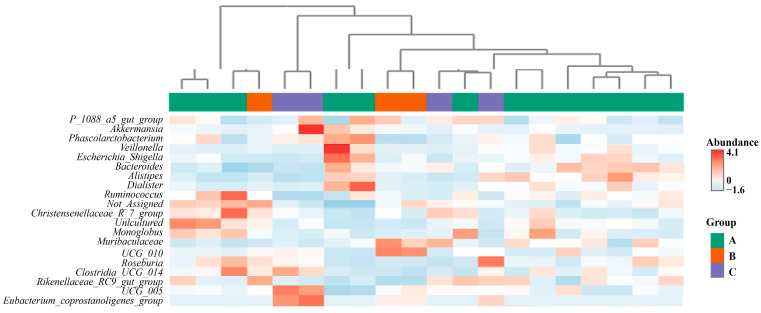
Cluster heatmap analysis based on the bacterial composition of the top 20 genera. Horizontal clustering represents the similarity of genera richness in the samples from Group A, B, and C. The color gradient from red to blue indicates relative abundance from high to low.

To visualize the detailed distribution of the gut microbiota at the genus level in the SMD across Groups A, B, and C, a heatmap depicting the top 20 genera was generated (Figure 8). In the heatmap, colored blocks represent the abundance of a specific genus in each sample. Horizontal clustering suggested no clear differences in the gut microbiota among the three groups. The heatmap revealed intra-individual differences at the genus level, irrespective of the sampling location of the SMD feces.

**Figure 9:**
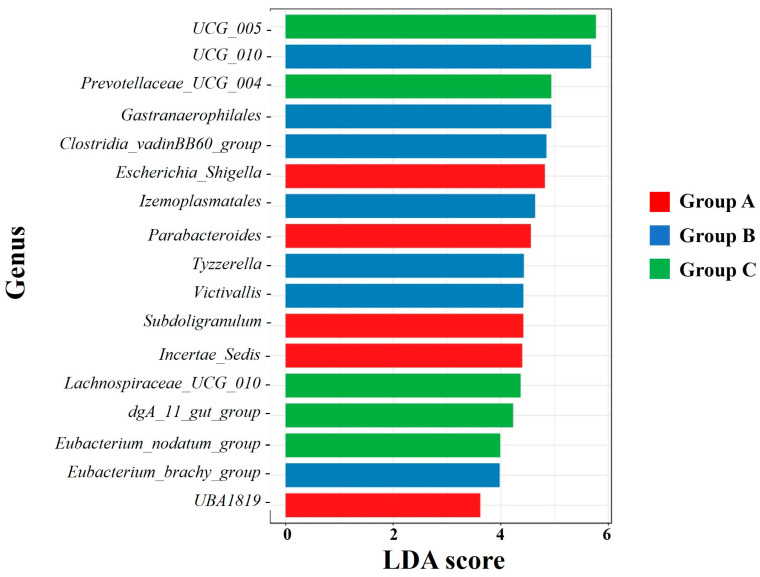
Linear discriminant analysis (LDA) histogram identifying significantly different taxa among Groups A, B, and C. The length of the bar column represents the LDA score (LDA > 2).

LEfSe, a tool for identifying specific microbiota with significant differences among groups, was used to detect differences at the genus level among Groups A, B, and C. Seventeen bacterial genera were identified to have a high relative abundance in specific groups, with an LDA score > 2. *Escherichia_Shigella*, *Parabacteroides*, *Subdoligranulum*, *Incertae_Sedis*, and *UBA1819* exhibited a considerably higher abundance in Group A than in Groups B and C; and *Oscillospiraceae*_*UCG_010*, *Gastranaerophilales*, *Clostridia_vadinBB60_group*, *Izemoplasmatales*, *Tyzzerella*, *Victivallis*, and *Eubacterium_brachy_group* were more abundant in Group B than in Groups A and C. Conversely, *Oscillospiraceae*_*UCG_005*, *Prevotellaceae_UCG_004*, *Lachnospiraceae_UCG_010*, *dgA_11_gut_group*, and *Eubacterium_nodatum_group* were significantly more abundant in Group C than in the other groups (Figure 9).

**Figure 10:**
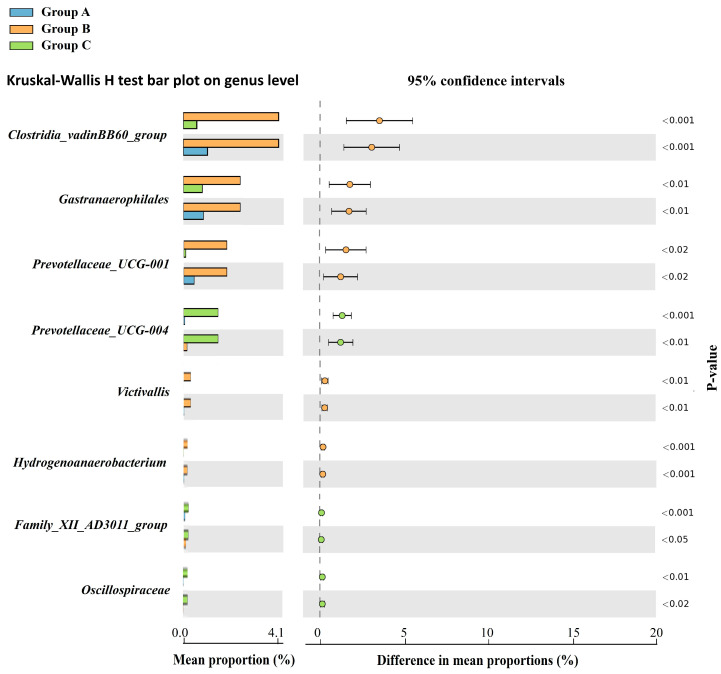
Histogram illustrating the differential abundance of taxa at the genus level among the three groups (A, B, and C).

Furthermore, taxonomic differences among the three groups were compared using STAMP v 2.1.3, to illustrate the genera with significant abundances within each group based on the Kruskal–Wallis H-test. Eight bacterial genera were identified to have a high relative abundance in specific groups (*p* < 0.05). The relative abundances of *Clostridia_vadinBB60_group*, *Gastranaerophilales*, *Prevotellaceae_UCG-001*, *Victivallis*, and *Hydrogenoanaerobacterium* were higher in Group B, whereas those of *Prevotellaceae_UCG-004*, *Family_XII_AD3011_group*, and *Oscillospiraceae* were higher in Group C than in the other groups (Figure 10).

## 4. Discussion

In this study, we analyzed the gut microbiota of wild SMD and compared data obtained from three different locations in Korea. To the best of our knowledge, this study represents the first analysis of the fecal microbiological composition of wild SMD. The findings of this study indicate that Firmicutes and Bacteroidetes are the predominant phyla in wild SMD, constituting approximately 90% of the microbiota, which is consistent with findings from existing research on the gut microbiota of ruminants, including AMD and FMD [16,18,40]. Firmicutes play a crucial role in ruminants by enzymatically degrading cellulose into volatile fatty acids [41], whereas Bacteroidetes primarily support gut immune functions by breaking down polysaccharides and proteins [16]. This dichotomy is reflected in the F/B ratio, which is an indicator of obesity in humans when elevated [42], and a marker of feed efficiency in ruminants [43]. A higher F/B ratio is associated with increased energy absorption and storage capacity [44] and positively correlates with the short-chain fatty acid (SCFA) concentrations [2]. Our study investigated the F/B ratios among the three groups (A: 2.30; B: 2.61; and C: 3.66), indicating a non-significant trend towards higher SCFA concentrations in Group C, followed by those in Groups B and A. Similarly, the relative abundances of Firmicutes and Bacteroidetes did not differ significantly among the three groups. These results indicate that habitual factors do not substantially affect the predominant phyla in the SMD.

Remarkably, Planctomycetota was identified as the third most abundant phylum across all the three groups (Figure 4A). Notably, Planctomycetota is not prominent in the gut microbiota of other musk deer species, including captive SMD, according to previous research [2,16,18,19]. Planctomycetota is associated with the secretion of carbohydrate-active enzymes, facilitating the degradation of lignocellulose [45] and chitin [46]. In goats, Planctomycetota is a prevalent phylum, particularly in the jejunal region, with abundances ranging from 5.3–15.9% [47]. Furthermore, a comparative analysis of yak and cattle microbiomes revealed significantly higher levels of Planctomycetota in yak rumen fluid than those in cattle [48]. These findings suggest a potential host-specific association between Planctomycetota and the gut microbiota of SMD, necessitating further investigation in future studies.

The relative abundance of Proteobacteria was significantly higher in Group A than in Groups B and C (Figure 7A). Proteobacteria are a well-known potential diagnostic criterion for dysbiosis, metabolic disorders, and inflammatory bowel disease (IBD) [49]. A low-fiber diet or acute/chronic inflammation disrupts the homeostasis of the host, causing the excessive proliferation of Proteobacteria in the gut [50]. Additionally, Proteobacteria and Firmicutes exhibit a competitive relationship, with Proteobacteria predominating at a young age (e.g., Pekin ducks for the initial three days, and piglets for one month) and Firmicutes becoming the dominant phylum with host maturity [51,52]. Consistently, the proportion of Firmicutes was lower in Group A (59.34%) than in Groups B and C (65.82% and 64.98%, respectively). Therefore, we hypothesized that Group A may include SMD with either a relatively unhealthy status or younger age than those in Groups B and C.

At the genus level, certain genera exhibited significant differences in relative abundance among the different groups. *Oscillospiraceae*_*UCG005*, a bacterial genus involved in the degradation of cellulose or hemicellulose [53], was significantly more abundant in Group C (22.00%), compared to Group A (10.37%) (Figure 7B). *Oscillospiraceae*_*UCG005* exhibits a positive correlation with acetate and total SCFA concentrations [54], suggesting that the SCFA concentration was the highest in Group C, followed by Groups B and A. This finding aligns with the results obtained for the F/B ratio.

*Prevotellaceae UCG-004*, identified as dominant in Group C using both LDA and the Kruskal–Wallis H-test, is associated with the antioxidant capacity of ruminants through the upregulation of ascorbate and aldarate metabolism [55]. Conversely, *Clostridiales vadin BB60*, which was significantly enriched in Group B, produces butyrate, which mitigates the risks associated with IBD, including ulcerative colitis [56]. *Escherichia-Shigella*, an opportunistic pathogen under the phylum Proteobacteria causing intestinal disorders [57], showed a higher abundance in Group A than in Groups B and C, as determined using LDA. These findings suggest that Group A may include SMD who are predisposed to gut diseases.

In the present study, an average of 17.9% of the total sequences were not assigned to any known genus (Figure 4B). Thus, the gut microbiome of SMD includes several unknown bacteria, which is consistent with other microbiome studies of FMD and AMD, where approximately 27.82% and 35.70% of the sequences were not classified into specific genera [16,18]. The gut microbiota of musk deer exhibited greater bacterial diversity, warranting further research to identify the unclassified sequences.

The alpha diversity analysis revealed significant differences in the gut microbiota richness among the three groups. Group A exhibited the highest total number of microbial species, followed by Groups B and C. However, the beta diversity analysis did not show significant differences among the groups at different locations. This result contradicts the findings of Pannoni et al. [8], who demonstrated that biogeography significantly affects the microbiome of wild hosts. There are two potential explanations for these results. First, the unequal distribution of samples among Groups A, B, and C, with fewer samples in Groups B and C, may have contributed to the low *R*^2^ value (0.22) observed in PCoA. Although an equal sample size is essential for accurate analysis, achieving this was challenging because of the biased habitats of wild SMD. Second, it is possible that the actual dietary habits among the three groups did not differ significantly owing to similar environmental conditions. Diet is a well-established primary factor directly influencing the diversity of the animal gut microbiota [58,59]. Studies on musk deer support this phenomenon, highlighting dietary factors as key contributors to the differences observed between species, captive and wild populations, and seasonal variations [2,16,18,19]. Hence, it is plausible to infer that the dietary patterns of SMD in Groups A, B, and C were analogous, leading to indiscernible disparities in the dominant gut microbiota. This finding aligns with the similar F/B ratios observed in this study. Meanwhile, in a previous study on captive SMD [2], the F/B ratio (ratio of 5.65–10.49) was considerably higher than in our findings (ratio of 2.30–3.66). Although a direct comparison cannot be made due to the differing methodologies, this variation may be related to diet. The primary diet of captive SMD in Mongolia consisted of Abietoideae and *Usnea* lichen in winter, and *Acer ginnala* and *Tricholoma mongolicum* in summer, whereas the diet of wild SMD in Korea remains largely unknown. Future research is needed to integrate the dietary analysis and food web surveys of wild SMD to obtain a comprehensive perspective on the microbiome results.

This study revealed structural differences in the gut microbiota among SMD inhabiting different locations. While no significant differences in gut microbiota diversity were observed among the groups, SMD in Group A exhibited significantly higher community richness than Groups B and C. To our knowledge, our study presents the first profile of the wild SMD gut microbial community, offering valuable insights into the microbiome-based monitoring of SMD conservation efforts.

The limitations of this study include the uneven sample distribution among the three groups. The small sample sizes in Groups B and C may affect the statistical power and reliability of our findings, potentially limiting the generalizability of the results. For a more accurate comparison of the gut microbiota between SMD in different regions, it will be necessary to obtain additional samples for Groups B and C to ensure uniformity in the sample size. Furthermore, investigating the dietary composition of SMD and the surrounding vegetation will provide a comprehensive assessment of the gut microbiome differences. In this study, 3 of the 23 samples were potentially compromised by external factors (e.g., weather), leading to the degradation of microbial DNA. Therefore, when conducting microbiome studies using fecal samples, it is advisable to collect fresh samples for prompt analysis.

## 5. Conclusions

This study represents the first comprehensive analysis of the fecal microbiome of wild SMD in distinct locations within the Gangwon Province, Korea. These findings highlight the composition and diversity of gut microbiota in these elusive and threatened wildlife species, offering crucial insights for their conservation and management. Despite significant differences in the gut microbiota richness among the study groups, the diversity analysis did not reveal any significant differences in the microbial composition between them. Nonetheless, the abundances of certain phyla and genera exhibited significant differences among the groups, indicating potential variations in habitat, diet, and health status. Overall, this study underscores the importance of microbiome-based monitoring in SMD conservation efforts and provides a scientific benchmark for assessing the health status of wild populations. Additional research that integrates dietary analysis and environmental factors is needed to clarify the complex interactions influencing the gut microbiota of wild SMD and to guide specific conservation strategies.

## Data Availability

The data presented in this study are available upon request from the corresponding author due to privacy concerns related to the geographic location.

## Supplementary Materials

The following supporting information can be downloaded at: https://www.mdpi.com/article/10.3390/ani14203000/s1, Table S1: Comparison of α-diversity indices of gut microbiota among Groups A, B, and C. Table S2. Relative abundances (mean ± SD) of (A) five major bacterial phyla and (B) nine major bacterial genera in Groups A, B, and C.
