# Supplementary material for: Insights into the Gut Microbial Diversity of Wild Siberian Musk Deer (Moschus moschiferus) in Republic of Korea
Source: Animals (Basel). 2024 Oct 17;14(20):3000. doi: 10.3390/ani14203000 (PMC11503724; doi:10.3390/ani14203000)
Supplement: Supplementary file 1 [file animals-14-03000-s001.zip › animals-3217091-supplementary.pdf]

Supplementary Table S1. Comparison of α-diversity indices of gut microbiota among Group A, B, and C

Alpha diversity index	Group A	Group B	Group C	*P*-value
ACE	358.37 ± 69.94*	305.22 ± 95.85	243.60 ± 36.79*	0.041
Chao1	358.65 ± 69.93*	305.75 ± 99.76	244.08 ± 35.66*	0.039
Shannon	4.91 ± 0.29	4.61 ± 0.37	4.43 ± 0.37	0.063
Simpson	0.98 ± 0.01	0.98 ± 0.01	0.97 ± 0.02	0.204

* Denotes groups exhibiting significant differences from Group A.
ACE: Abundance–based Coverage Estimator

Supplementary Table S2. Relative abundances (mean ± SD) of (A) five major bacterial phyla and (B) nine major bacterial genera in groups A, B, and C

(A)

**Phylum**	**Group A** (%)	**Group B** (%)	**Group C** (%)	***P*-value**
Firmicutes	59.34 ± 11.06	65.82 ± 4.01	64.98 ± 12.05	0.361
Bacteroidota	30.78 ± 8.06	25.72 ± 4.31	24.52 ± 13.47	0.386
Planctomycetota	3.58 ± 2.43	4.11 ± 2.99	5.56 ± 2.39	0.328
Proteobacteria	3.09 ± 1.99	0.94 ± 0.37	0.98 ± 0.91	0.032
Verrucomicrobiota	0.95 ± 1.10	1.32 ± 0.86	2.82 ± 4.10	0.626

(B)

**Genus**	**Group A** (%)	**Group B** (%)	**Group C** (%)	***P*-value**
Bacteroides	16.37 ± 0.08	10.15 ± 0.05	11.52 ± 0.05	0.34
Oscillospiraceae_UCG_005	10.37 ± 0.04	13.93 ± 0.03	22.00 ± 0.1	0.02
Oscillospiraceae_UCG_010	3.19 ± 0.02	12.47 ± 0.06	4.52 ± 0.02	0.02
Eubacterium_coprostanoligenes_group	3.98 ± 0.02	2.84 ± 0.01	8.28 ± 0.04	0.02
Muribaculaceae	3.57 ± 0.02	8.05 ± 0.02	3.35 ± 0.03	0.08
p_1088_a5_gut_group	3.58 ± 0.02	4.11 ± 0.03	5.56 ± 0.02	0.33
Christensenellaceae_R_7_group	3.64 ± 0.03	3.32 ± 0.01	3.54 ± 0.02	0.94
Rikenellaceae_RC9_gut_group	3.38 ± 0.02	2.42 ± 0.04	3.55 ± 0.02	0.65
Ruminococcus	2.94 ± 0.02	2.00 ± 0.00	1.89 ± 0.01	0.21
